# The feasibility of a train-the-trainer approach to end of life care training in care homes: an evaluation

**DOI:** 10.1186/s12904-016-0081-z

**Published:** 2016-01-22

**Authors:** Andrea Mayrhofer, Claire Goodman, Nigel Smeeton, Melanie Handley, Sarah Amador, Sue Davies

**Affiliations:** Centre for Research in Primary and Community Care (CRIPACC), University of Hertfordshire, Hatfield, Hertfordshire AL10 9AB UK; Marie Curie Palliative Care Research Department (MCPCRD), University College London (UCL), formerly Centre for Research in Primary and Community Care (CRIPACC), University of Hertfordshire, Hertfordshire, UK

**Keywords:** End of life care, Training, Care homes, Programme implementation

## Abstract

**Background:**

The ABC End of Life Education Programme trained approximately 3000 care home staff in End of Life (EoL) care. An evaluation that compared this programme with the Gold Standards Framework found that it achieved equivalent outcomes at a lower cost with higher levels of staff satisfaction. To consolidate this learning, a facilitated peer education model that used the ABC materials was piloted. The goal was to create a critical mass of trained staff, mitigate the impact of staff turnover and embed EoL care training within the organisations. The aim of the study was to evaluate the feasibility of using a train the trainer (TTT) model to support EoL care in care homes.

**Methods:**

A mixed method design involved 18 care homes with and without on-site nursing across the East of England. Data collection included a review of care home residents’ characteristics and service use (*n =* 274), decedents’ notes *n =* 150), staff interviews (*n =* 49), focus groups (*n =* 3), audio diaries (*n =* 28) and observations of workshops (*n =* 3).

**Results:**

Seventeen care homes participated. At the end of the TTT programme 28 trainers and 114 learners (56 % of the targeted number of learners) had been trained (median per home 6, range 0–13). Three care homes achieved or exceeded the set target of training 12 learners. Trainers ranged from senior care staff to support workers and administrative staff. Results showed a positive association between care home stability, in terms of leadership and staff turnover, and uptake of the programme. Care home ownership, type of care home, size of care home, previous training in EoL care and resident characteristics were not associated with programme completion. Working with facilitators was important to trainers, but insufficient to compensate for organisational turbulence. Variability of uptake was also linked to management support, programme fit with the trainers’ roles and responsibilities and their opportunities to work with staff on a daily basis.

**Conclusion:**

When there is organisational stability, peer to peer approaches to skills training in end of life care can, with expert facilitation, cascade and sustain learning in care homes.

## Background

Residents in care homes are in the last years of life and often present with multiple health needs, cognitive impairment, and particular palliative care needs due to their advanced age [1]. The implementation of education and training targeted at end of life (EoL) care is, therefore, particularly important for those working in long term care [2–4]. The challenge is how to equip and sustain the workforce to provide generalist palliative care in settings where the staff have limited access to specialist services, many do not have a formal qualification, and turnover of staff is high [5, 6].

In October 2012 NHS Health Education East of England (formerly East of England Multi-professional Deanery) commissioned a local specialist palliative care service to develop the Train the Trainer (TTT) End of Life Care Education Programme for care home staff. This built on the success of the ABC End of Life Education Programme that had trained approximately 3000 care home staff across the East of England in EoL care [7]. When the ABC programme was compared with another EoL training framework (Gold Standards Framework)[8] for care homes it achieved equivalent outcomes in terms of impact on staff satisfaction, confidence and competence, and in satisfaction of next-of-kin [8]. The ABC programme was preferred by participants, because trainees felt they were personally supported by the visiting nurse specialists in palliative care [9]. It was also considered to be the more cost effective of the two schemes reviewed [8] and reported a modest reduction in death rates in hospital, unscheduled admissions and bed days.

**Fig. 1:**
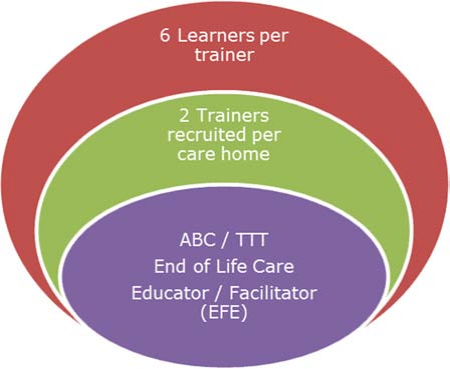
TTT Team configuration between EFEs, trainers and learners

Building on the ABC programme, and working with the same network of specialist palliative care services, the Train the Trainer (TTT) project aimed to train two ‘trainers’ per care home, who in turn were to train six ‘learners’ each (*n =* 12 per care home). In order to become a ‘trainer’ one had to have participated in the ABC programme, which consisted of six EoL care training modules, some input pertaining to learning and teaching methods, and practice workshops with EoL care educators/facilitators (EFEs). Trainers’ responsibilities included the preparation of on-line and face-to-face teaching sessions, the organisation and facilitation of group discussions, and ideally offering learners bite-size micro-teach sessions in daily practice. Full teaching sessions were observed and evaluated by End of Life Care Educators/Facilitators (EFEs). The EFEs were employed by a range of organisations and held various clinical and education roles including palliative link nurse, palliative care nurse, practice-development nurses for care homes, EoL care specialist and EoL educator. The configuration that underpins the TTT model process is depicted in Fig. 1.

The goal of the TTT pilot project was to consolidate the success of the ABC EoL care programme, increase the capacity of the care home workforce to provide EoL care, and develop a model that could sustain training in and provision of EoL care in care homes. The pilot ran for nine months (Oct 2012–June 2013). The research questions focused on what supported or hindered the uptake of the programme. Number of learners trained was used as a proxy measure by which to judge the TTT model’s effectiveness in embedding and extending the knowledge and practice of EoL care across care homes.

## Methods

Eighteen care homes across three counties in the East of England were recruited to the project. Thirty six care home staff, who had completed ABC training, were selected to be ‘trainers’ in EoL care. Recruitment of individual care homes was based on criteria such as their endorsement of the programme, geographical fit with existing palliative care services, their previous engagement with ABC EoL care training and identification of staff who had completed ABC training and could take on the role of trainer. As the evaluation of the TTT training model was commissioned after the programme had been implemented a before-after study design was not possible.

### Data collection

A mixed method design using qualitative and quantitative data was used. Quantitative data consisted of Service Use Logs and data collected using modified InterRAI forms [10] from a 30 % randomly selected sample of residents (*n =* 274) in participating care homes. These data were used to establish a baseline of resident characteristics and care requirements, and to estimate resource use such as visits from primary care services and admission to hospital. Resident Service Use data were collected for three months from April to June 2013. The study also reviewed care notes of residents who had died (*n =* 150) post intervention, between October 2012 and July 2013, to establish if previously observed findings from the ABC evaluation of advance care planning (ACP), documentation of palliative care, symptom management and place of death were sustained [8, 9]. Findings from data collected via care notes have been reported elsewhere [9].

To understand the implementation process of the TTT model semi-structured face to face interviews (*n =* 39) were conducted (Table 1), and focus groups (*n =* 3) were held with trainers, EFEs, a project lead and care home managers.

**Table 1:** Number of interviewees per care home

Study site	Trainers	Learning facilitators (EFEs)	Managers	Total
S1	10	4	1	15
S2	11	4	0	15
S3	6	2	1	9
Total	27	10	2	39

Trainers were also given audio diaries to record reflections and experiences arising from their role, but staff found these diaries cumbersome to use. The yield of data was negligible. Data collection took place from October 2012 until the end of July 2013. Participating care homes granted permission to collect audit data, and written consent was obtained from all interviewees.

### Data analysis

Quantitative variables were summarised by medians, ranges and percentages. The number of learners trained in each care home was compared by type of ownership (for profit organisation versus not for profit organisation), type of care home (residential versus on-site nursing), size of the care home (less than 60 versus 60 or more residents), and previous and/or additional EoL care training (yes versus no), using the Mann–Whitney *U* test. Statistical analyses were performed using SPSS [11].

Qualitative data collected in interviews were recorded, transcribed, anonymised and analysed using QSR NVivo Version 10 [12]. Qualitative data analysis involved cross-sectional and categorical indexing across care homes to enable comparisons. Three researchers were involved in the analysis of data. The study was approved by the National Institute for Social Care and Health Research (REC 12/WA/0384). Social Care Research Governance Approval was obtained from Local Authorities (LAs).

## Results

Three of the 18 eligible care homes left the programme soon after it commenced. In two care homes staff were no longer available to attend the training workshops and in the other the reasons were unknown. Two further care homes were recruited as replacements, which resulted in a total of 17 participating care homes. Of 34 trainers (two per care home) 28 completed the three skills training workshops to support their trainer role. All trainers had completed the ABC training and held a variety of roles, ranging in seniority from General Manager to Support Worker, including Care Home Trainers who held responsibilities for all mandatory training, but were not directly involved in caring for residents (Table 2).

**Table 2:** Roles of trainers by site

Study site	Role of trainer 1	Role of trainer 2
S1	Trainer in Care Home	Carer
S1	General Manager	Carer
S1	Carer	Care Team/Unit Manager
S1	Nurse	Carer
S1	Clinical Manager	Receptionist
S1	Carer	Care Team/Unit Manager
S2	Care Team/Unit Manager	Only 1 trainer
S2	Deputy Manager	Carer
S2	Deputy Manager	Care Team/Unit Manager
S2	Care Team/Unit Manager	Carer
S2	Care Team/Unit Manager	Carer
S2	Care Team/Unit Manager	Night Unit Manager
S3	Trainer in Care Home	Only 1 trainer
S3	Carer	Only 1 trainer
S3	Nurse	Nurse
S3	Trainer in Care Home	Only 1 trainer
S3	General Manager	Deputy Manager

Learners recruited were care home staff with similarly varying levels of seniority. At the end of the TTT project 114 learners had been trained (median per home 6, range 0–13). Three care homes achieved or exceeded the set target of training 12 learners. Two care homes had not trained any learners at the end of the pilot.

This variability was investigated in relation to care home and resident characteristics to see if the uptake of the programme might have been linked to factors such as (for care homes) how a care home was funded, on site nursing provision, size of care home, and how many staff had already received EoL care training, and (for residents) the presence of individuals with more complex health care needs or shorter life expectancies.

### Care home characteristics

Table 3 reflects type of care home, type of ownership, and additional training by county.

**Table 3:** Type of care home, type of ownership, and additional training by site

	Site 1 (*n =* 6)	Site 2 (*n =* 6)	Site 3 (*n =* 5)
Care home residential	2	5	2
Care home with on-site nursing	4	1	3
Care home ‘for profit’	6	0	4
Care home ‘not for profit’	0	6	1
Additional EoL Care training (Gold Standards Framework) completed or in progress	5	1	1

More learners were trained in care homes owned by for profit organisations (median = 7.5) than in care homes owned by non-profit organisations (median = 5), but there was no statistical evidence for a difference (*p =* 0.475, Mann–Whitney *U* test). The comparisons of the median number of learners by type of care home (residential = 7.5, nursing = 5.0: *p =* 0.423), size of care home (less than 60 residents = 7.5, 60 or more = 6.0: *p =* 0.888), and previous or additional EoL care training in some care homes (yes = 9.0, no = 5.5: *p =* 0.475) were also not statistically significant.

### Resident characteristics and resource use

Table 4 presents summary information on the residents and their use of health care resources. The number of learners trained in each care home could have been influenced by differences in the resident population of participating care homes and the services received. For example, residents in some care homes might have needed more support from visiting health care professionals than residents in other care homes or been identified as approaching the end of life. However, the sample of 274 residents fitted the national profile of care home residents in terms of gender, cognitive ability, co-morbidities and function as indicated in the literature [13, 14]. Literature does not report any association of these factors with care home staff engagement. Likewise, based on the qualitative data in this study there was nothing to suggest that residents’ characteristics or care needs influenced whether a care home was more or less likely to engage with the programme.

**Table 4:** Resident characteristics and resource use

Age at admission (years) median (range)	83 (38–99)
Female (%)	189/254 (74.4)
Diagnosis of dementia (%)	166/252 (65.9)
Condition reaching end-stage (%)	34/250 (13.6)
Advance Care Planning in place (%)	116/225 (51.6)
No admission to hospital (%)	227/238 (95.4)
Total GP visits, median (range)	1 (0–10)
Palliative care visit (%)	3/268 (1.1)

### Factors influencing programme uptake

As discussed in the following section, the qualitative data suggested that the variation in uptake was attributable to three key contextual factors. These were the role and responsibilities of trainers within the care home, the uptake of EFE facilitation by the care home, and the stability of the care home in terms of leadership and staff.

#### Trainers’ professional roles and responsibilities

As indicated in Table 2, trainers’ professional roles varied greatly, and this determined their opportunities to spend time with learners during programme implementation. Where teaching could be integrated with patterns of working there was a greater likelihood of staff engagement and discussion. For example, the teaching impact seemed greater when a ‘trainer’ and a ‘learner’ worked on the same unit and had opportunities to discuss the application of theory to ‘real life’ situations. As expressed by a trainer:

*“…if we know that someone is very near EoL we discuss every aspect i.e. what we are going to do, what the care plans say, what they [the residents] need, do they need mouth care, what’s working for them, what pain relief they are on… so we do a catch-up session and pre-plan what we are going to do in relation to all the topics we have covered”* [Trainer, experienced carer, T01011].

Due to staff shifts it was often difficult to get six individuals together for group work at the same time. Trainers were encouraged by EFEs to adapt their support of learners to reflect the preferences of individuals and the working patterns of the care homes. This required a level of flexibility and autonomy that was not always possible because of the trainer’s role and other commitments in the care home.

The ability to incorporate the trainer’s role into the existing work schedule also had an impact on the uptake of the TTT programme. When trainers held managerial posts, this often meant that they had to create time to carry out training within the specified timeframe, as it was difficult to use routine encounters with staff and residents as opportunities for learning and review. As expressed by one of the managers who acted as trainer:

“*This is extra to my job and time consuming*” [Trainer T01051SA].

This was also commented on by an EFE (training facilitator, palliative care specialist) who concluded:

*“…if I were to choose a care home [to participate in a TTT EoL care education and training intervention] I would be thinking very carefully about the manager and the person who is going to be the trainer [in relation to] what their other commitments are. It has been very difficult to work with a trainer who is managing a unit and has numerous other responsibilities going on. You need to make sure you have someone with passion [for EoL care] and dedicated [ring-fenced] time to become involved in training learners”* [E0205].

In addition, not all learners were equally ready to receive training at a particular level. For example, some less experienced care staff found it difficult to watch emotionally challenging content about death and dying on DVDs on their own. They preferred group work and discussions that could offer immediate debriefing. As stated by a trainer, the ability to be present during learning helped to address emotional reactions to the training:

*“…some emotional issues were dealt with during training (in relation to talking about death); this was an opportunity to discuss how they could/would best support each other…”* [T01011].

Not only trainers in relation to learners, but also Educators/Facilitators (EFEs) in relation to trainers were aware of this critical part of EoL care training. As one palliative care specialist emphasised:

*“An EFE role needs to be in place for mentorship debrief, support, and on-going training …”* [E0207].

This was also a critical part of post-training support until staff had formed their own support groups within care homes.

#### Uptake of EFE facilitation by care homes

The TTT programme did not specify how EFE facilitators should work with care homes. Care homes’ different uptake of facilitator training support is shown in Table 5.

**Table 5:**
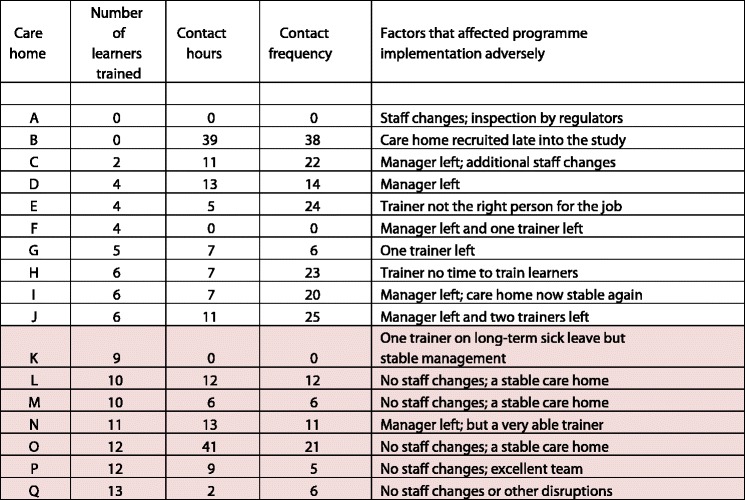
Number of learners trained by EFE input and care home stability

Significantly more learners were trained in stable care homes (median = 10.5) than in those that were not stable (median = 4.0) (*p =* 0.001, Mann–Whitney *U* test). This study defined care home stability in terms of staff changes during project implementation where staff were involved in the project either as trainers, learners, facilitators or managers. As shown in Table 5, care home ‘B’ had considerable EFE input, but did not train any learners. This care home was recruited late into the study and the trainer, a relatively junior member of staff, received intensive support to compensate for not having been able to attend an earlier workshop. This improved her confidence, skills, and recognition in the care home as someone knowledgeable in end of life care, but had not translated into peer to peer education by the end of the pilot project. In contrast, care home ‘O’ also shows high levels of contact hours, but in this case contact time was related to the supply of additional reading material and subsequent discussions around improving practice. High levels of EFE input were used to supplement existing expertise rather than to compensate for limited access to prior support.

Care homes ‘A’, ‘F’, and ‘K’ had no recorded EFE input. Care home ‘A’ experienced critical staff changes and an inspection by regulators early on during project implementation. Trainers did not attend their second workshop, therefore could not teach any learners and consequently required no EFE input. In care home ‘F’ the specialist facilitator left her post just after the pilot project had started. The role was not replaced, so there was no specialist EFE input during programme implementation. Four learners completed the on-line EoL care training modules, but their learning was not assessed. In care home ‘K’ one of the trainers went on long term sick leave during project implementation. The remaining trainer did not engage with the EFE facilitator, but asked her learners to watch the online modules. Nine learners completed the modules, but the learning had not been assessed at the end of the pilot programme. However, the EoL care specialists remained available for post-training support, and it is likely that the learners’ knowledge would have been assessed at a later stage. Finally, care home E shows a relatively high contact frequency (24 attempts), but only 5 h of input. The trainer’s unsuitability for the role referred to the fact that the EFE’s repeated attempts to engage with the trainer, and suggested ways of working together, had been unsuccessful. The trainer had not volunteered for the role and found it difficult to engage with End of Life care training.

The intention at the outset of TTT was that staff involvement would be voluntary and that those with an explicit commitment, following on from their ABC training to become trainers, would take up the role. However, there were few opportunities for staff to discuss timing and the level of involvement with their EFEs before the commitment to participate had been made. As one project lead pointed out,

*”… capacity constraints in some care homes meant that we were asking people [trainers and learners] to participate who were not self-selected…”* [EPL01].

The ramifications of non-self-selection were seen in the care homes where training uptake was lower, or where EFEs’ repeated attempts to keep in touch with trainers were unsuccessful or limited as indicated in Table 5.

Within the TTT programme post-training support is conceptualised as a continuing relationship between trainer, learner and an EFE, until newly trained staff have reached a new level of confidence in administering EoL care to residents in their care. Both trainers and EFEs stated that post training support was one of the most important aspects of the Train the Trainer model and key to its success. The use of EFE facilitators alone however did not predict uptake of the programme, and could not compensate for trainers who had not volunteered to participate, or for disruptions in the care home environment.

#### Care home stability in terms of leadership and staff

Key to the uptake of the training programme was the intrinsic stability of the care home. All seven of the ‘high achieving’ care homes, where high achieving is defined by numbers of learners trained, benefited from a stable environment during programme implementation, whereas nearly all of the other care homes had adverse circumstances to cope with (Table 5). A stable environment in this study refers primarily to staff involved in the training programme, for example managers, trainers or learners, not leaving during programme delivery. The seven care homes which had trained between nine and 13 learners (shaded in Table 5) benefited from senior management support for the programme, and from little or no observable staff changes during project implementation, particularly of managers, trainers and learners. Conversely, care homes that trained about half the number of learners originally aimed at were characterised by managerial change, temporary lack of leadership, and trainers and/or learners leaving. In six of the care homes the manager left whilst the EoL care programme was implemented, which meant that other staff, including some ‘trainers’, had to deputise. In three of the care homes either one or both trainers left. The capacity of the care homes to participate had not been assessed at the beginning of the programme. Their previous engagement with the ABC programme, which had focused on individual learning, had been the sole criteria for participation. In hindsight one facilitator commented:

*“…the difference between the [care] homes where training has worked is where you’ve got strong managers and strong leaders, and I imagine a certain amount of stability”* [E0207].

## Discussion

Despite the limitations faced in different care homes the TTT programme successfully trained 114 care home staff across 17 care homes within nine months. The TTT approach demonstrated the feasibility of introducing a supported care home based peer to peer education model for end of life care.

A combination of factors influenced project implementation, training uptake and numbers of learners trained in this study. Whilst organisational commitment, staff interest and access to expert facilitation were important, they were not sufficient to guarantee uptake. A care home’s readiness to participate in an intervention or not at a particular point in time was shaped by the roles and responsibilities of trainers and where they were located in the care home structure, the opportunities for learning that occurred as part of the everyday workflow, and the level of organisational turbulence in terms of staff turnover and sustained engagement of senior management.

Organisational readiness for change is a well-known construct that embraces multiple organisational/structural and psychological/cultural determinants at various levels [15]. Some of the organisational/structural factors which influenced the uptake of training and therefore numbers of learners trained in this study include managerial stability or the lack thereof [16], supportive management [17], lack of staff involvement in preparing for programme implementation [18–21], capacity constraints that impacted on the release of staff for training [16, 22], and post training support through collegiate working [23, 24].

Whilst the issue of care home stability in relation to staff turnover is recognised and discussed in international literature [25–28], this does not often translate into how EoL care interventions are planned. There is considerable heterogeneity in the care home sector that needs to be taken into account from the outset. There is an increasing recognition of the need to consider what kind of facilitation works best in care homes and with which staff groups [29]. As observed in previous research [30], and supported by these findings, increased facilitation and hours of training are not sufficient to overcome the negative impact of factors such as trainer availability and selection, and organisational flux.

Effective programme implementation relies on an understanding of organisational structure, leadership and staff interaction, and readiness for change *prior* to implementing interventions [31–33]. This is not to imply that care homes should only be recruited for training in end of life care once they are ‘ready’. What is needed is a structured approach that can be used to highlight which organisational factors need to be addressed, not only to prepare to engage with the training programme, but also to benefit from the intervention. This would need to involve a pre-intervention phase of discussion and review between care home and palliative support staff to identify facilitators and barriers to an EoL care intervention, favoured (and achievable) learning styles, and how to assess the level of facilitation that might be required.

### Implications for policy and practice

Findings indicate that the Train the Trainer model has the potential to build and sustain the capacity of the workforce to deliver end of life care to older people dying in care homes. However, the implementation of an EoL care training programme will, depending on care home readiness, require different levels and types of external facilitation and support. The process of assessing care home readiness itself can serve to build commitment to project participation [19–21], and thereby influence training uptake and outcomes, particularly if prospective trainers and learners are invited to be part of the pre-assessment process.

A raised awareness of the concept of organisational readiness for change can contribute significantly to implementation planning and begin to explain why some care homes are more able than others to implement and to sustain EoL care programmes over time. A raised awareness of the concept of organisational readiness for change also has resource implications, as programme implementation at the wrong time could be minimised by pre-assessing the capacity of a care home to accommodate and/or engage with an intervention at a particular point in time.

## Limitations

A limitation of this evaluation is that participating care homes were pre-selected and limited to one geographical region. In addition, the study could not offer pre-post measures, because the intervention had already commenced the implementation process when its evaluation was commissioned. It is also likely that findings were influenced by various existing initiatives in some care homes.

## Conclusion

A peer education model for end of life care training, with facilitation, in care homes is feasible and well placed to equip and sustain the workforce to provide a high standard of EoL care. However, a structured approach to the assessment of care home readiness to engage with EoL care education and training interventions is necessary. This should involve the mapping of enablers and barriers, and the planning of strategies for facilitation and resource allocation.
